# Prevalence, correlates and pattern of hepatitis B surface antigen in a low resource setting

**DOI:** 10.1186/1743-422X-8-12

**Published:** 2011-01-12

**Authors:** Ahizechukwu C Eke, Uzoamaka A Eke, Charles I Okafor, Ifeanyichukwu U Ezebialu, Chukwuanugo Ogbuagu

**Affiliations:** 1 Masters in Public Health, Harvard School of Public Health, 677 Huntington Avenue, Boston, Massachusetts, USA; 2 Dept of Infectious Diseases, University of Connecticut Health Center, 263 Farmington Avenue, Connecticut, USA; 3 Dept of Obstetrics and Gynecology, Nnamdi Azikiwe University Teaching Hospital, Nnewi, Anambra State, Nigeria; 4 Accident & Emergency Dept, Nnamdi Azikiwe University Teaching Hospital, Nnewi, Anambra State, Nigeria

## Abstract

**Background:**

Hepatitis B virus (HBV) infection in Nigeria has remained a Public Health issue. It is a major cause of mortality, especially in developing countries. Vertical transmission of hepatitis B virus infection is thought to be a major route of transmission in low resource areas. In spite of this, routine antenatal screening for hepatitis B infection is not yet practiced in many Nigerian hospitals. This paper present the findings of a study conducted among antenatal women in Nnewi, Nigeria.

**Methods:**

It was a cross-sectional study carried out over a 3-month period (August - October, 2009). Recruitment of 480 women attending antenatal clinics in Nnewi, Nigeria was done by simple random sampling using computer generated random numbers. HBsAg screening was done using rapid ELISA Kits. Statistical analysis was computed using STATA 11 package. The results were subjected to analysis using cross tabulations to explore statistical relationships between variables. Chi square test was used to explore proportional relationship between groups. The level of statistical significance was set at p < 0.05 (providing 95% confidence interval).

**Results:**

Four hundred and eighty pregnant women were recruited into the study. Of these, 40 tested positive to HBsAg, accounting for 8.3% of the sample population. The age of the subjects studied varied from 14 to 45 years (mean age - 24.3 years) while the mean parity was 2.18. The HIV/HBV co-infection rate was 4.2%. The vertical transmission rate was 51.6%. There were statistically significant relationships between HBV infection and previous history of tribal marks/tattoos (χ^2 ^= *27.39, P = 0.001, df = 1)*, history of contact with previously infected HBV patients (χ^2 ^= *23.11, P = 0.001, df = 1) *and occupation of the women (χ^2 ^= *51.22, P = 0.001, df = 1)*. Multiple sexual partners, blood transfusion, dental manipulations, sharing of sharps/needles, and circumcision were not significant modes of transmission. There was no statistically significant relationship between maternal age, educational level and HBV infection.

**Conclusion:**

The authors argued that hepatitis B screening in pregnancy should be made routine practice in Nigeria because of the low pick up rate of the infection based only on risk factors for the disease.

## Introduction

Infection with hepatitis B virus (HBV) is a serious global public health problem [1-3]. In the continents of Africa and Asia, it remains a major cause of morbidity and mortality [1-3]. Annually, up to 1 million of these infected population die due to the consequences of the infection such as liver cirrhosis and hepatocellular carcinoma [4]. Despite the existence of a safe and effective vaccine, Nigeria has remained a hyper-endemic area for hepatitis B virus infection, with an estimated 12% of the total population being chronic carriers [5]. Hepatitis B virus infection continues to be a substantial and devastating health problem, with new cases still being reported annually [6,7].

The prevalence of HBV infection among an antenatal population may be a reliable indicator of hepatitis B virus prevalence rate in the general population. Hence, screening antenatal women for HBsAg can give a reliable prevalence of the disease in a population and provide an avenue for preventing mother to child transmission of the virus. A Demographic Health Survey (DHS) by the World Health Organization (WHO) in 2003 showed that in the developing countries of Africa, 68% of women report at least one ante-natal visit [8]. This study by the World Health Organization reported that at least 70% of women reported for antenatal care four or more times in Nigeria [8]. 74% of these women who accessed antenatal care in Nigeria are from the urban areas while 44% are from the rural areas [8]. The study showed that women presenting for antenatal care in Nigeria are most likely to wait until the second trimester and a relatively substantial proportion present only in the third trimester [8]. It has also been shown that vertical transmission of hepatitis B virus occur in up to 10% of neonates when the infection occur in the first trimester and in 60% to 90% of babies in the third trimester [9].

Sero-prevalence studies on HBsAg in Nigeria have shown that the prevalence of the infection in pregnant women range from 2-15% [10-13]. Screening for HBsAg is routine in pregnancy in most developed countries of the world. It is a recommendation of the Royal College of Obstetricians and Gynaecologists (RCOG) of the United Kingdom [14], the American College of Obstetrics and Gynecology (ACOG) [15], and most of the other 122 Colleges of Obstetrics and Gynecology all over the world to screen pregnant women for hepatitis B surface antigen routinely in pregnancy. However, in Nigeria, screening of antenatal women for hepatitis B virus is not a routine practice. Also, routine vaccination of newborns is not widely available in low resource settings in Nigeria. Even though Nigeria approved the inclusion of hepatitis B vaccine in its National Program on Immunization (NPI) in 1995, the vaccine only became widely available in 2004 [16]. Despite the fact that the vaccine had been made available via the NPI, immunization coverage for Hepatitis B is still not optimal in low resource settings in Nigeria [16]. A recent study in Nigeria showed that the Hepatitis B vaccine coverage rate in low resource settings is currently about 41% [17]. The consequences of the problems of low pick up rate of HBV infection due to poor screening and the low vaccination rate are that vertical transmission of hepatitis B virus has become the major route of transmission of the virus in Nigeria. This has a major impact on the babies when born because of the risk to developing chronic liver diseases later in life.

Acute hepatitis in pregnancy has been shown to induce premature labour and prematurity with its attendant effects [9,18]. Studies have shown an increase in the incidence of prematurity over that seen in the general population [9,18,19]. The Apgar scores were also shown to be lower in the newborn of HBsAg carriers, and there was a higher risk of intra-ventricular hemorrhage as well, which was probably related to the higher incidence of preterm delivery [19]. Apart from this, intra-partum and post-partum hemorrhage from coagulation failure due to lack of production of vitamin K dependent clotting factors, especially if the prothrombin time is prolonged as in fulminant hepatic failure, are problems that could present following chronic hepatitis B virus infection. Again, in utero infection with Hepatitis B virus remains very important because some fetuses that contact the hepatitis B antigen early in embryonic development become immunologically tolerant to the antigen, leading to a chronic HBV infection caused by the inability to eliminate the virus [18].

Prevention of vertical transmission is extremely important because HBV infection in early life can result in a chronic carrier state. As shown in earlier studies, administration of hepatitis B immunoglobulin (HBIG) to HBsAg-positive mothers reduced the rate of vertical transmission to 23%, and the combination of HBIG and hepatitis B vaccination reduced transmission even further to 3% [18,20]. Passive-active immuno-prophylaxis with hepatitis B immunoglobulin (HBIG) and hepatitis B vaccine in the infants of HBV carriers gives high levels of protection against vertical transmission [18]. Administration of HBIG in a dose of 200 international units, intramuscularly every week from 28th weeks of gestation reduces mother to child transmission of hepatitis B virus [9,18,21]. Lamivudine, a cytosine analogue that acts as a nucleoside reverse transcriptase inhibitor, is a potent inhibitor of HBV replication [22]. Lamivudine therapy leads to rapid reduction of HBV-DNA levels, with a median reduction of 97% after 2 weeks [9]. It has been used with good safety and efficacy in the last four weeks of pregnancy to decrease the risks of vertical transmission[9,22]. Li et al, in 2003 showed that intrauterine infection was reduced to 16.3% against 32.7% (controls) with the use of lamivudine from the 28th week of gestation at a dose of 100 mg/day [23]. The role of Caesarean section in reducing mother to child transmission of HBsAg has been studied and it was found that it does not show any extra reduction in the incidence of immunoprophylaxis failure in comparison to vaginal delivery. There is no evidence that Caesarean section prevents maternal-infant transmission of hepatitis B virus [9,14,18].

Since hepatitis B virus infection has serious sequelae, there is a continuing need to examine its epidemiology, especially in low resource settings in Nigeria. The Objective of this study therefore, was to determine the prevalence, correlates and pattern of Hepatitis B surface antigen among pregnant women attending antenatal clinics at the Nnamdi Azikiwe University Teaching Hospital, Nnewi, Nigeria. This centre is also dedicated to the Prevention of Mother To Child Transmission of HIV (PMTCT), so we handle a significant number of HIV positive pregnant women as well. Therefore, the study compared the prevalence of Hepatitis B surface antigen among the HIV sero-negative and HIV sero-positive patients.

## Methods

It was a cross-sectional study, conducted at the Nnamdi Azikiwe University Teaching Hospital, Nnewi, Nigeria. Nnamdi Azikiwe University Teaching Hospital, Nnewi is a center for the Prevention of Mother to Child Transmission of HIV (PMTCT) for the south eastern geo-political zone in Nigeria. The study was carried out among pregnant women attending antenatal care at the Nnamdi Azikiwe University Teaching Hospital, Nigeria.

The research was conducted after approval was obtained from the ethics and research committee of the hospital. The study recruited 480 pregnant women attending antenatal clinics at the Nnamdi Azikiwe University Teaching Hospital, Nnewi by simple random sampling using computer generated random numbers. The sample size was derived using the formula for sample size calculation: N = z^2^pq/d^2 ^(where z = Standard normal deviation at 1.96 (which corresponds to 95% confidence interval; p = Prevalence of Hepatitis B surface antigen in ante-natal women in Nigeria from previous studies; q = 1-p; and d = degree of accuracy/precision expected = 0.05.

The subjects who met the criteria for inclusion into the study were recruited after an informed consent was obtained from the subjects. The inclusion criteria for the study included any pregnant woman who gave consent. Subjects who did not give consent were excluded from the study. The main outcome measures were the detection of HBV carriage status by detecting the presence of Hepatitis B surface antigen in the sera of pregnant women and associations between HBsAg status and known predisposing factors, including demographic and clinical predictors for maternal infection. Neonatal HBV infection was also an outcome measure.

The subjects then completed an anonymous questionnaire on the predisposing factors to hepatitis B virus infection following a literature review. The confidentiality of information in the questionnaire was expressed in the introductory part of the questionnaire. The questionnaires were distributed in an envelope, with an explanatory letter assuring anonymity. There was no information on the questionnaire that permitted identification of individual respondents. The self-completed questionnaires required the subjects to fill out information on demographics, knowledge about hepatitis B virus and its screening and risk factors for hepatitis B virus infection. Risk factors assessed included previous history of blood transfusions, number of sexual partners in the past, previous history of contact with people infected with hepatitis B virus, history of tattoos/tribal marks and previous history of surgical or dental procedures. The questionnaires were initially pre-tested on 63 pregnant women for comprehensiveness, effectiveness, reliability and validity. A number of minor changes were subsequently made.

After completion of the questionnaire, five milliliters (5 mls) of blood sample was collected from the subjects after tourniquet application. To obtain plasma, venous blood was collected into Ethylene Di-amine Tetra-acetic Acid (EDTA) bottles and plasma separated for HBV assay by centrifugation (using a centrifuge) at 6,000 revolutions per minute (rpm) for 5 minutes. Hepatitis B surface antigen determination was then done with rapid testing kits by applying the plasma onto the test kits.

Parallel testing for HBsAg was used for testing. Parallel testing involves the use of two rapid Enzyme Linked Immuno-Sorbent Assay (ELISA) test kits simultaneously. The two rapid testing kits used in the study were the hepatitis B surface antigen ELISA kits produced by Antek diagnostics Incorporated, United Kingdom, and Acumen diagnostic kits (Acumen diagnostics Incorporated), China. The use of two or more kits was based on the WHO recommendation of at least two different testing strategies (algorithms) involving simple rapid assays for surveillance. A portion of plasma from each of the subjects screened was dropped on the specimen pad on one end of both test strips simultaneously and a waiting time of 15 minutes was observed before the results are read. Two simultaneously positive test results were interpreted as positive. If one result was positive and one negative, a third kit was used as a tie breaker to resolve this difference (Acon Hepatitis B ultra fast diagnostic kits by Acon Biotech diagnostic systems, United States of America).

The strips were pre-coated with anti-hepatitis B surface antibodies (anti-HBs) on the test line region. During the experiment, the plasma reacted with the particle coated with anti-HBs antibodies. The mixture migrated on the membrane chromatographically by capillary action and reacted with anti-HBs antibodies that generated a colored line. After 15 minutes, in the positive samples, distinct red lines appeared in the control region (C) and another line in the test region (T). In the negative samples, only one red line appeared in the control region (C). The test strips were read in collaboration with a Medical laboratory technologist. The results were interpreted as follows: Positive (1^st ^kit), Positive (2^nd ^kit) - positive result; Negative (1^st ^kit), Negative (2^nd ^kit) - negative result; Positive (1^st ^kit), negative (2^nd ^kit) - subject to a 3^rd ^test kit (tie-breaker). If positive - positive result, if negative - negative result; Negative (1^st ^kit), Positive (2^nd ^kit) - subject to a 3^rd ^test kit (tie breaker). If positive - positive result, if negative - negative result.

The data was assessed for normality using box plots. Statistical analysis of the results was computed using STATA 11 package. The results were subjected to analysis using cross tabulations to explore statistical relationships between variables. Equality of variances between groups was assessed before independent two-sample t test for equal and unequal variances was used for comparison of the means. Chi square test was used to explore proportional relationship between groups. The level of statistical significance was set at p < 0.05 (providing 95% confidence interval).

## Results

During the 3 months study period, 480 women were enrolled into the study. Of these, 40 tested positive to HBsAg, accounting for 8.3% of the sample population. The age of the women studied varied from 14 to 45 years (mean age - 24.3 years) - (Table 1). The mean parity was 2.18. More than half - 264 (55.0%) of the women had secondary level education while 124 (25.8%) and 44 (9.2%) had primary and tertiary education respectively - (Table 1).

**Table 1:** Socio-demographic characteristics of the women

*Characteristic*	*Number (n)*	*Percent (%)*
10-14	4	0.8
15-19	72	15.0
20-24	124	34.2
25-29	88	18.3
30-34	80	16.7
35-39	56	11.7
40-44	12	2.5
45-49	4	0.8
		
***Educational level***		
Primary	124	25.8
Secondary	264	55.0
Tertiary	44	9.2
No formal education	48	10.0
		
***Religion***		
Christianity	472	98.3
Islam	8	1.7
		
***Ethnicity***		
Igbo	456	95.0
Tiv	4	0.8
Yoruba	8	1.7
Igala	4	0.8
Urhobo	8	1.7
		
***Occupation***		
Healthcare provider	36	7.5
Non health care provider	444	92.5
		
***Marital Status***		
Single	20	4.2

Married	460	95.8

HBsAg was detected in 40 (8.3%) out of 480 women. None of them were previously aware of their condition. There were no maternal or neonatal deaths. The prevalence of HBsAg was highest among the 20-24 age group (table 2), where 20 women out of the 40 tested positive to HBsAg. The mean gestational age was 24.08 weeks. The mean age, parity and gestational ages were not statistically significant between the HBsAg - positive and sero-negative women. Fifty six (11.9%) of the women sampled were HIV positive. Out of the 56 women who tested positive to HIV, 20 (35.7%) of the respondents who were HIV positive were also positive to HBsAg. This was not statistically significant *(P = 0.402)*. The HIV/HBV co-infection rate from the study was therefore (20/480 or 4.2%).

**Table 2:** Age and HBsAg status of the pregnant women

*Age group*	*(Number)*	*HBsAg (+)*	HBsAg (-)
**10-14**	4	0 (0.0%)	4 (100.0%)
**15-19**	72	12 (16.7%)	60 (83.3%)
**20-24**	164	20 (12.2%)	144 (87.8%)
**25-29**	88	0 (0.0%)	88 (100.0%)
**30-34**	80	8(10.0%)	72 (90.0%)
**35-39**	56	0 (0.0%)	56 (100.0%)
**40-44**	12	0 (0.0%)	12 (100.0%)
**45-49**	4	0 (0.0%)	4 (100.0%)

X^2 ^= 6.237; df = 7; P = 0.512

One hundred and thirty two (27.5%) women gave a history of contact with a relative, a spouse, a friend or someone with hepatitis B infection in the past, while 348 (72.5%) had never had contact with anyone with hepatitis in the past (Table 3). Among those who had a history of contact with someone with hepatitis B, 24 people tested positive to HBsAg. This was statistically significant (χ^2 ^= *23.11, P = 0.001, df = 1)*. There was no history of hepatitis B vaccination in any of the subjects. The presence of body inscriptions like scarification marks or tattoos were statistically significant routes of transmission of HBV (χ^2 ^= *27.39, P = 0.001, df = 1)*. - (Table 3). Previous history of blood transfusion, history of previous dental or surgical procedures and previous history of circumcision/use of sharps were not statistically significant routes of transmission of HBV.

**Table 3:** Predisposing factors to HBsAg

Variables	(Number)	HBsAg (+)	HBsAg (-)	**X**^**2**^	P-value
**Multiple sexual partners**	480				
-*Yes*	140	12(11.4%)	124(88.6%)	0.168	0.682
-*No*	340	24 (7.1%)	316 (92.9%)		

**Previous history of Surgery/Dental procedure**	480				
-*Yes*	112	12 (10.7%)	100 (89.3%)	1.084	0.298
-*No*	368	28 (7.6%)	340 (92.4%)		

**Previous history of Blood transfusion**	480				
- *Yes*	80	0 (0.0%)	80 (100%)	2.182	0.140
*- No*	400	40 (10.0%)	360 (90.0%)		

**History of Circumcision**	480				
*- Yes*	96	8 (8.3%)	88 (91.7%)		
*- No*	252	28 (11.1%)	224 (88.9%)	1.851	0.396
*- I don't know*	132	4 (3.0%)	128 (97.0%)		

**History of tribal marks/tattoos**	480				
-*Yes*	20	8 (40.0%)	12 (60.0%)	27.39	0.001
-*No*	460	32(7.0%)	428 (93.0%)		

**History of contact with hepatitis B infected patient in the past**	480				
- *Yes*	132	24 (18.2%)	108 (81.8%)	23.11	0.001
*- No*	348	16 (4.6%)	332 (95.4%)		

**Previous history of sharing of sharps/needles**	480				
*- Yes*	320	32 (10.0%)	288 (90.0%)	3.490	0.062
*- No*	160	8 (5.0%)	152 (95.0%)		

The women's occupation as a risk factor to the acquisition of HBsAg was also reviewed. There were 36 healthcare workers and 444 non healthcare workers sampled. Out of the 36 healthcare workers, 8 (22.2%) tested positive to HBsAg. This was statistically significant (χ^2 ^= *51.22, P = 0.001, df = 1)*. The eight women that tested positive to HBsAg were midwives at maternity centers.

Educational attainment was also considered as a risk factor for hepatitis B virus transmission. Ninety percent of the women studied had at least elementary education. Those that attained up to secondary education comprised the majority of the subjects - 264 (55.0%). Among the 264 women with secondary education, 20 (7.6%) tested positive to HBV, while only 12 (9.7%) were positive among those with primary education and 4(9.1%) at the tertiary level respectively. This was not statistically significant (χ^2 ^= *0.5247, P = 0.769, df = 2*).

Regarding parity and hepatitis B virus infection from the study, multigravidae (89.2%) comprised the majority of subjects compared to primigravidae (10.8%). When the two were compared against the risk of infection with hepatitis B infection, there was no statistically significant relationship between them (χ^2 ^= *2.244, P = 0.814, df = 1)*.

Married subjects comprised the majority of subjects screened for HBsAg (460 cases or 95.8%). However, though the rest of the subjects were grouped as 'single', some of these were actually co-habiting with their sexual partners. When marital status was considered as a predisposing factor to HBV infection, it was not statistically significant (χ^2 ^= *0.8109, P = 0.368, df = 1)*.

When the impact of not breastfeeding a baby when a mother was HBV positive was assessed, 436 - (90.8%) of the mothers stated that they would be sad if asked not to breastfeed on account of HBV positivity. Twenty (4.2%) said it would be okay not to breastfeed and 24 women (5%) were indifferent about breastfeeding in a HBV sero-positive state. Infant feeding options were discussed with the women that tested positive to HBsAg and 32 of them opted for breastfeeding.

All the 40 women that screened positive to hepatitis B surface antigen (HBsAg) had never been screened for HBsAg before. Also, majority of the women who screened negative to HBsAg - 432 (91.5%) had never been screened before. Eight women who screened negative to HBsAg had screened negative previously. On the willingness to be screened again in subsequent pregnancies, those with no formal education were significantly unwilling to come again versus those with education. This was shown to be statistically significant (χ^2 ^= *61.37, P = 0.001, df = 1*). Curiously enough, among those with education, the more highly educated women were less likely to be willing to come for re-testing.

## Discussion

The results of this study showed that the prevalence of HBsAg among pregnant women attending the antenatal clinic in this low resource setting was 8.3%. Hence, according to the WHO classification for Hepatitis B endemicity, Nnewi is a hyper-endemic area for hepatitis B virus infection. This prevalence is identical to the study at the Ahmadu Bello University Teaching Hospital Zaria (ABUTH), Nigeria - 8.3% [24]. It is however, higher than the prevalence of 2.2% from a study in Benin-city, Nigeria [25]. It is also higher than the reported prevalences of 4.3% in Port-Harcourt [26], 4.4% in Lagos [10] and 4.6% in Enugu [27], all in Nigeria. However, results from other parts of Nigeria and Africa showed higher prevalence of HBsAg. These include a prevalence of 9.3% in Awka [11], 11% in Makurdi [12] and 15.8% at the University of Maiduguri Teaching Hospital (UMTH), Maiduguri, Borno state, Nigeria [13].

In most epidemiological studies on HBsAg, there has been a link between age and the acquisition of HBsAg. The age of acquiring the infection is one of the major determinants of the prevalence rates of HBsAg. In this study, HBsAg was highest among the 20-24 age group (Table 2). A similar finding was noted in the study by Vazquez-Martinez et al who observed that the average age of women infected with the Hepatitis B virus in Mexico was 26 years [28]. The findings from this study did not tally with the findings from the study by Habiba et al, where majority of those that tested positive to HBsAg were in the age range of 25-35 years [29]. The reason for this may be because this is the age group in which most women in South-Eastern Nigeria are most likely to get married and become pregnant. It is also the age when they are likely to present for the first time for ante-natal care. Hence, those positive to HBsAg are likely to be picked up when screened as was noted from this study.

From the study, there was a high prevalence of HBsAg among the pregnant women, yet, only few of the known predisposing factors to hepatitis B virus infection showed statistical significance. A similar result was obtained by Summer et al when they showed that most of those that tested positive to HBsAg had one or two predisposing factors to hepatitis B surface antigen [30]. It was noted from this study that previous histories of tribal marks/tattoos, occupation of the subjects and previous contact with somebody with hepatitis infection were statistically significant predisposing factor to HBsAg infection. The risk of transmission of HBV has been known to be high in people who are in contact with chronically infected subjects [6,31,32].

Another possible explanation for the high prevalence of HBsAg among pregnant women in Nnewi, Nigeria is that the infections could have resulted from vertical transmission. The vertical transmission rate from this study was 51.6%. The vertical route is a major mode of transmission of the virus [6,7,25]. In a study done in Benin City, Nigeria, it was shown that the vertical transmission rate was 42.9% for HBsAg, which was a very high rate of transmission, similar to the rate from this study [25]. Multiple sexual partners, previous history of sexually transmitted infections, previous histories of blood transfusion and previous surgeries/dental manipulations were not statistically significantly associated with HBV infection from this study. This is in keeping with the study in Port-Harcourt, Nigeria [26].

Out of the 56 women who tested positive to HIV, twenty (35.7%) also tested positive to HBsAg. This was shown not to be statistically significant. The HIV/HBV co-infection rate from this study was 4.2%. This rate is higher than the 0.7% HIV/HBV co-infection rate among pregnant women screened in Awka, Nigeria [10], but less than a co-infection rate of 11.5% in Jos, Nigeria [33]. The result of this study corroborated with some other studies in Africa that have shown HIV/HBV co-infection to be low. A study in Malawi by Ahmed et al showed that there was no statistical evidence to suggest that HIV positivity was associated with an increased prevalence of HBV [34]. Another study among pregnant women attending ante-natal clinics in Burkina Faso showed a low co-infection rate of 0.88% [35]. HIV/HBV co-infection was also very low (0.20%) in a study in Benin- city, Nigeria where it was concluded that obstetric co-infection with HIV and HBV was a rare phenomenon in their parturients [25].

Another important factor studied was the link between the level of education of the pregnant women and the acquisition of HBV as well as the willingness to be re-tested for HBsAg in subsequent pregnancies. Ninety percent of the women studied had at least elementary education. This finding was in keeping with the study in Mexico, which showed that the educational status of the population was high since 78% of the women studied had at least elementary education [28]. It was also noted that the education of the women appeared to improve respondent's willingness to accept testing and re-testing in subsequent pregnancies, be it primary, secondary or tertiary education. However, those with no formal education were significantly unwilling to come again versus those with education. Curiously enough, among those with education, the more highly educated one was, the less likely they were willing to come for re-testing. The reason for this may be that those with tertiary education felt that testing once for the HBsAg was enough irrespective of whether or not they were repeatedly exposed to predisposing factors to HBsAg. They might think that re-testing would be a waste of time. Hence, from this study, it is important to educate women on the need for repeated testing, at least once in every pregnancy during antenatal care sessions.

Occupation of the subjects is a known predisposing factor for HBsAg infection [36]. From the study, eight out of the 36 health care workers (22.2%) screened positive to HBsAg. This was statistically significant. It is expected that pregnant health care workers are supposed to know about the modes of transmission of HBsAg and thus, protect themselves from getting infected. However, those in high risk health departments like blood transfusion services or theatres may be more predisposed to acquiring the infection compared to health care workers in pharmacies, out-patient departments and administrative departments of hospitals. The fact that the eight pregnant women that tested positive to HBsAg were midwives at maternities goes further to buttress the point. As midwives, they were exposed to body fluids from women during delivery and possibly acquired the infection from body fluids from these women at delivery. This study also showed expectedly that health workers were significantly more likely to have heard about screening for HBsAg, compared to their non health care counterparts.

The study also showed that majority of the mothers (97%) wanted to breastfeed their babies even when infected with HBV. There is evidence that HBV can be transmitted from infected mothers to their babies through breast milk [18,19,21]. Therefore, avoidance of breastfeeding lowers the risk of HBV transmission from an infected mother to the baby. On the other hand, non breastfed infants are prone to serious health risks, including diarrheal disease and other infections in infancy. Infant feeding options were discussed with the women that tested positive to HBsAg and 32 of them opted for breastfeeding.

There were some limitations to this study. The study was done in a single low resource centre in Nigeria. Even though the study was done in a single low resource center, it is a very big referral center for urban and especially, rural pregnant women in South-eastern Nigeria. So, it is a good place to do research because of the diversity of patients managed in this center. It was also a cross-sectional study. In future, when funds become available, a prospective multi-centre study would be carried out to further validate our findings. The strength of our study is in the fact that it was carried out in a low resource region not previously studied. The study also highlights the major routes of transmission of the hepatitis B virus in this low resource setting in Nigeria, and the current challenges as regards routine screening of women in pregnancy, and vaccinating newborns immediately after delivery. The study was able to show that vertical transmission of the virus was the commonest route of transmission in low resource settings in South-eastern Nigeria.

## Conclusion

The results of this study highlighted the high prevalence and correlates of HBsAg in Nnewi, Nigeria and its pattern among pregnant women attending antenatal care. Despite the high prevalence of HBsAg from the study, there was a low pick up rate based only on predisposing factors to hepatitis B virus. The high prevalence underscores the need for preventive measures against hepatitis B virus infection in pregnant women. It also underscores the need for routine testing of the virus in pregnant women during antenatal sessions. With increased campaign for testing, it is believed that more success will be made in this area. Thus, it is imperative that women be properly educated by healthcare personnel on the various routes of transmission of the virus, the infant feeding options available and the need for screening and re-screening for HBsAg in pregnancy. There is also need for treatment of positive cases to prevent mother to child transmission of HBV.

## Conflict/Disclosure of interests

The authors declare that they have no competing interests.

## Authors' contributions

AE, UE, CO, IE and CO were all involved in the study conception and design, acquisition of data, analysis and interpretation of data, drafting of manuscript and the critical revision of the manuscript. All authors read and approved the final manuscript.
